# Learning curve analysis of artificial intelligence-assisted ultrasound for teaching popliteal fossa sciatic nerve block to novice anesthesiologists: a pilot randomized educational study

**DOI:** 10.7717/peerj.21877

**Published:** 2026-11-12

**Authors:** Yun Wang, Yanmin Chen, Fu Chen, Junzuo Fu, Hong Tian, Jinbao Li, Chongzhi Zhou, Huiming Li, Lina Huang

**Affiliations:** 1 Shanghai General Hospital, Shanghai, China; 2 Department of Medical Education, Shanghai General Hospital, Shanghai Jiao Tong University, Shanghai, China; 3 Jiuquan Hospital of Shanghai General Hospital (Jiuquan People’s Hospital), Jiuquan, China; 4 The Shanghai Medical Doctor Association, Shanghai, China

**Keywords:** Artificial intelligence-assisted ultrasound, Ultrasound-guided regional anesthesia, Learning curve, Anesthesiologist training, Popliteal sciatic nerve block, Pilot randomized educational study

## Abstract

**Background:**

Artificial intelligence (AI)-assisted ultrasound may improve ultrasound-guided regional nerve blocks, but its effect on novice learning curves is unclear.

**Methods:**

In this pilot randomized educational study (June–August 2025), inexperienced anesthesiology residents from Shanghai General Hospital were assigned to AI-assisted or conventional training groups. The AI group used an ultrasound system with an AI module and received additional ethics and principles training, while the conventional group received standard training. Image acquisition and interpretation, debriefing time, trainee speaking time, and feedback were evaluated.

**Results:**

Twenty-one trainee anesthesiologists were recruited (AI, *n* = 10; conventional, *n* = 11). The group-level cumulative sum (CUSUM) analysis identified an estimated turning point (TP) at approximately 12 attempts in the AI group. No comparable TP was identified within the 15-attempt training period in the conventional group. No statistically significant between-group differences were detected in participant-level phase-specific success rates (all *P* > 0.05). Compared with the conventional group, the AI group reported higher post-training feedback scores for training content (4.7 ± 0.5 *vs*. 3.6 ± 1.0, *P* = 0.0076), satisfaction (4.9 ± 0.3 *vs*. 3.5 ± 0.8, *P* < 0.0001), and confidence (5.0 ± 0.0 *vs*. 4.5 ± 0.7, *P* = 0.0216). Compared with the conventional group, the AI group also had a shorter participant-level mean debriefing time (7.33 ± 0.34 *vs*. 10.65 ± 0.61 min, *P* < 0.0001) and a greater proportion of trainee speaking time during debriefing (69.5% (65.70, 70.55) *vs*. 49.0% (46.70, 49.70), *P* < 0.0001).

**Conclusions:**

AI-assisted ultrasound training might enhance post-training feedback, increase satisfaction and confidence, and reduce debriefing time among inexperienced anesthesiology residents, suggesting its potential utility in the medical education of ultrasound.

## Highlights

(1) An exploratory CUSUM turning point (TP) was identified within the observed training range in the AI group, whereas no comparable TP was observed in the conventional group
(2) No statistically significant between-group differences were detected in phase-specific success rates
(3) Artificial intelligence (AI) training reduced debriefing time and increased trainee speaking time during debriefing
(4) Trainees reported higher satisfaction, confidence, and training content scores

## Introduction

Ultrasound-guided regional nerve blocks have been widely recognized as the standard of care in perioperative anesthesia practice (Griffin & Nicholls, 2010). By enabling direct visualization of the target anatomy, needle trajectory, and spread of local anesthetics, ultrasound guidance significantly increases block success rates and reduces complication risks compared with traditional landmark-based or nerve stimulator-guided approaches (Lewis et al., 2015; Perlas et al., 2008), thereby improving analgesic efficacy (Gelfand et al., 2011) and patient safety (Jogie & Jogie, 2023) and lowering the minimum effective anesthetic volume required for successful blockade (Di Filippo, Falsini & Adembri, 2016). Despite its advantages, the successful implementation of ultrasound-guided regional nerve blocks is highly dependent on the operator’s skill (Chin & Chan, 2008), particularly in acquiring optimal images and accurately identifying the relevant anatomical structures (Swain et al., 2024). The development of these skills requires extensive hands-on practice and prolonged observation, making the learning process technically demanding and resource intensive (Barrington et al., 2012; Sites et al., 2010). Inadequate image acquisition ability or misinterpretation of critical anatomic structures may induce block failure, highlighting the need for targeted and effective training for clinicians. Nevertheless, the use of standardized training among clinicians remains low (Haskings et al., 2023), and individual proficiency in anatomy and ultrasound interpretation varies considerably (Bowness, El-Boghdadly & Burckett-St Laurent, 2021; Bowness et al., 2023a). These challenges, together with the high cost and time investment required for comprehensive training programs, have limited the broad delivery of high-quality ultrasound-guided nerve blocks in clinical practice.

The emergence of artificial intelligence (AI)-assisted image recognition has opened new possibilities for medical image analysis and interpretation (Bowness, El-Boghdadly & Burckett-St Laurent, 2021; Bowness et al., 2023a; Chan et al., 2020). By utilizing large, well-annotated datasets and advanced computational power, AI models can achieve performance comparable to that of experienced clinicians in various diagnostic and procedural tasks (Oren, Gersh & Bhatt, 2020; Sokol, Fackler & Vogt, 2025) while offering additional advantages such as consistency, efficiency, and scalability. In ultrasound-guided nerve blocks, AI-assisted image recognition has shown particular promise because it accurately identifies key anatomical structures and enhances nerve localization efficiency (Bowness, El-Boghdadly & Burckett-St Laurent, 2021; Bowness et al., 2021, 2023a; Bowness et al., 2022). In teaching settings, AI-assisted ultrasound systems may overcome challenges in conventional training, such as limited access to sufficient clinical cases, steep learning curves, and variability in instructor availability (Jacobs, Wainman & Bowness, 2024; Viderman et al., 2022). By providing real-time anatomical guidance and feedback, such systems have the potential to accelerate skill acquisition and improve training efficiency. Preliminary studies suggest that AI tools can improve the accuracy and confidence of novice operators, potentially transforming current training paradigms (Bowness et al., 2021, 2022, 2023b; McKendrick, Yang & McLeod, 2021). However, these observations are largely based on expert opinion, and robust evidence from specialized studies remains scarce.

In this study, we aimed to compare the impact of AI-assisted and conventional ultrasound systems on the learning curve of novice anesthesiologists in acquiring and interpreting ultrasound images for popliteal fossa sciatic nerve blocks.

## Methods

### Study design and participants

This was a pilot randomized educational study conducted among trainee anesthesiologists at Shanghai General Hospital between June and August 2025. The study was designed to generate preliminary evidence regarding the potential educational value of AI-assisted ultrasound for novice training in popliteal fossa sciatic nerve image acquisition and interpretation. Recruitment information was posted on the hospital’s internal bulletin board system, and the participants signed up voluntarily. The inclusion criteria were as follows: (1) anesthesiology residents in the standardized residency training program; (2) novice practitioners with less than 2 years of anesthesia experience; and (3) no prior training or practical experience in ultrasound-guided peripheral nerve blocks. The exclusion criterion was any physical or mental condition deemed unsuitable for participation in ultrasound-guided nerve block training by the supervising faculty. Healthy volunteers were recruited *via* an advertisement posted on the internal platform of the hospital. The inclusion criteria were as follows: (1) aged 18 years or above; (2) body mass index (BMI) between 18 and 28 kg/m^2^; and (3) intact skin over the popliteal fossa without lesions. The exclusion criteria were as follows: (1) history of allergy to ultrasound coupling agents; (2) history of knee joint or lower limb surgery; (3) pregnancy; and (4) anatomical abnormalities of popliteal vessels, nerves, muscles, or other structures, as confirmed by an experienced physician *via* ultrasound imaging. Individuals meeting these criteria were enrolled as ultrasound teaching models. This study was approved by the Ethics Committee of Shanghai General Hospital (Approval No. ylk[2025]238) and registered at the Chinese Clinical Trial Registry (https://www.chictr.org.cn/, Registration No. ChiCTR2500104731). Written informed consent was obtained from all the participating trainees and volunteers.

### Procedures

The study procedure is shown in Fig. 1. Prior to study initiation, all trainee participants completed a questionnaire recording their age, sex, training grade, and academic qualifications. All healthy volunteers completed a questionnaire recording their age, sex, and body mass index (BMI). To reduce selection bias, group allocation was performed using a computer-generated allocation sequence generated in SPSS Statistics 25 software (IBM, Armonk, NY, USA), and group assignments were concealed from trainee participants until the start of training. The recruited residents were allocated to either the AI-assisted ultrasound group (AI group) or the conventional ultrasound group.

**Figure 1:**
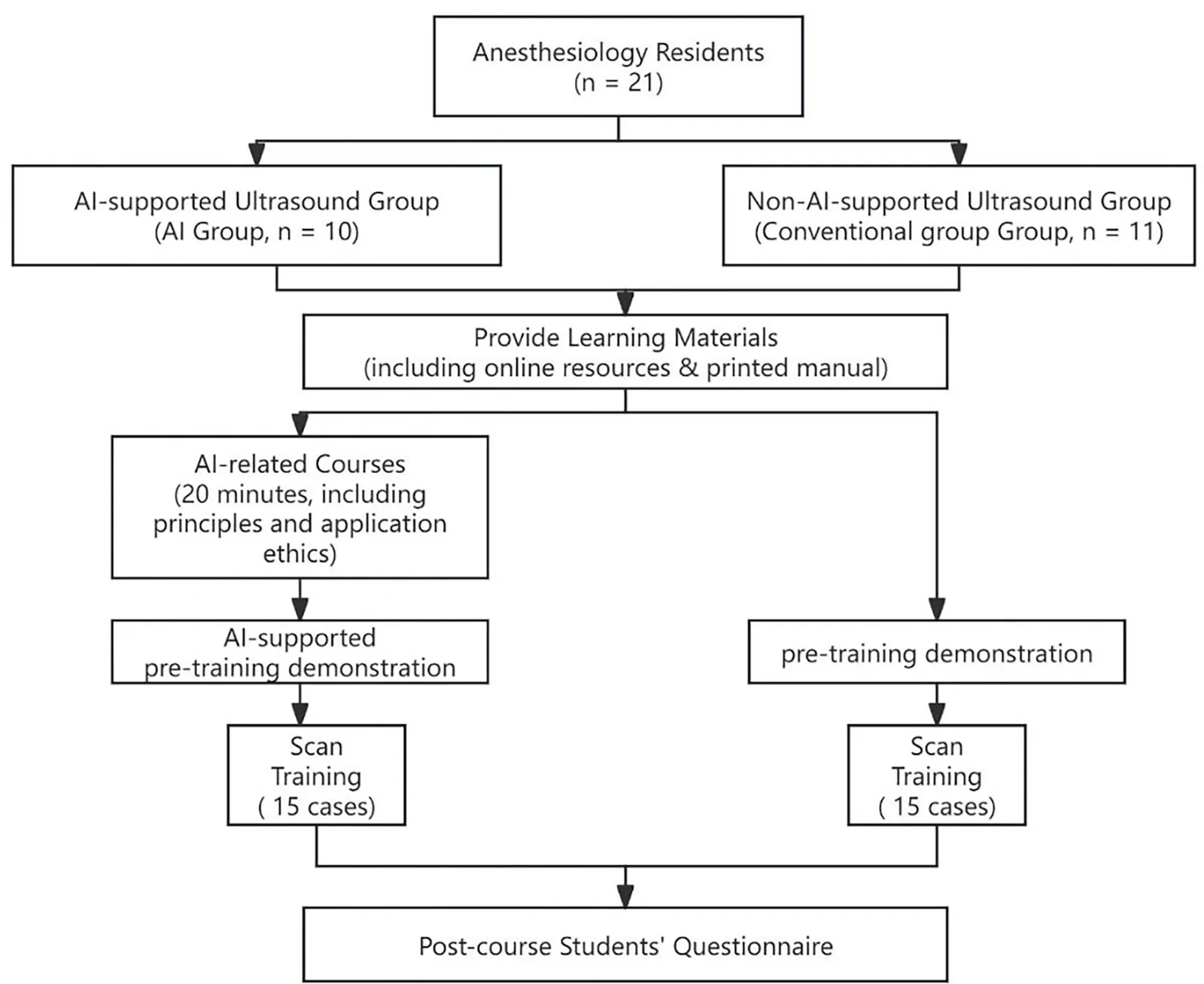
Flow diagram of this study.

Before the practical sessions, all trainee participants attended the same preparatory training, which included printed materials on the physical principles of ultrasound imaging and scanning techniques and an electronic learning module on sonoanatomy of the popliteal fossa for sciatic nerve block. The AI group received an additional 20-min online lecture on the technical principles and ethical considerations of AI-assisted ultrasound, which was delivered by faculty from the Shanghai Jiao Tong University School of Medicine.

Three days after completing the preparatory sessions, the residents were asked to participate in the practical training program. All the trainees used the same ultrasound platform (Labat Wisonic, Shenzhen Wisonic Medical Technology Co., Ltd., Shenzhen, China). Depending on allocation, the AI assistance function (WiNerve) was enabled for the AI group and disabled for the conventional group. The initial pretraining demonstration was delivered by the same senior anesthesiologist, who instructed all residents in acquiring and identifying the sciatic nerve and adjacent structures in the popliteal fossa *via* the ultrasound platform.

The practical training program consisted of 15 cases over three consecutive weeks. The 15 healthy volunteers were scheduled in a fixed sequence, and each volunteer was scanned by trainees from both groups. Trainees participated in a shuffled order within each session to ensure that both groups were exposed to the same volunteer-related anatomical conditions. For each volunteer, the trainee positioned the volunteer in the lateral decubitus position and prepared the ultrasound equipment. The trainee was then asked to place the linear high-frequency probe (LH15‒6) 3‒4 cm above the popliteal crease in the short-axis view and scan distally to visualize the sciatic nerve bifurcation into the tibial and common peroneal nerves, along with the popliteal artery and vein, biceps femoris, semitendinosus, and semimembranosus muscles (Fig. 2). The trainee was allowed to freely adjust the probe position, gain, and depth and use color Doppler; images could be frozen at any time for anatomical identification. During scanning, the instructor and assistants were not permitted to provide any verbal or physical guidance or hints. Once a trainee judged the target image to be optimal, the image was frozen, and the anatomical structures were identified verbally by the trainee. This process was recorded by the teaching assistant for subsequent evaluation.

**Figure 2:**
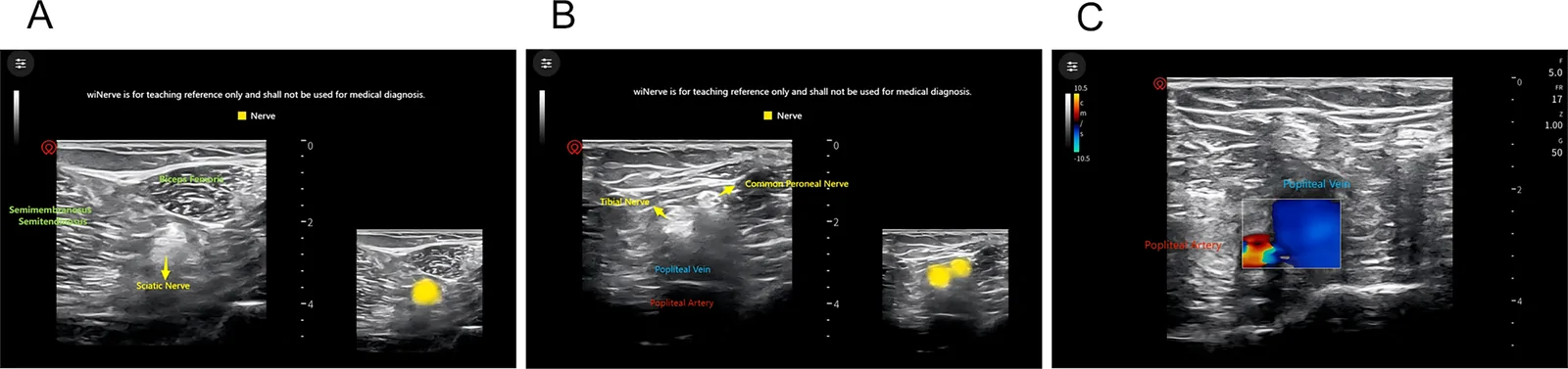
Example ultrasound images. (A) Ultrasound image of the popliteal fossa with the AI system enabled. The yellow overlay in the right panel highlights the sciatic nerve as identified by the WiNerve module. (B) Ultrasound image of the popliteal fossa with the AI system enabled. The yellow overlay in the right panel highlights the tibial nerve and common peroneal nerve as identified by the WiNerve module. (C) Ultrasound image of the popliteal fossa with the AI system disabled and Doppler mode activated.

The instructor and a teaching assistant, both mid-level anesthesiologists, independently evaluated each recorded attempt. A scan was classified as successful if the image was considered clear and all target structures (sciatic nerve, tibial nerve, common peroneal nerve, popliteal artery, and popliteal vein) were correctly identified; otherwise, it was classified as unsuccessful. Disagreements between the two assessors were resolved by a third experienced anesthesiologist through review of the recording. Since AI-generated anatomical overlays were visible in the recordings, the assessors could not be blinded to group allocation.

After each practical case, a one-to-one debriefing session was conducted to review errors and provide corrective feedback. Each debriefing session was video recorded, and the duration was documented. Upon completion of the 15-case series, all the residents completed an anonymous questionnaire evaluating their training experience.

### Outcomes and measurement

The primary outcome was the cumulative sum (CUSUM) learning curve of successful and unsuccessful ultrasound image acquisition and interpretation (Kollmann-Camaiora et al., 2017). At attempt i, the δ score of a trainee k was defined as

${\delta _{i,k}} = {X_{i,k}} - {X_o},$

where ${X_{i,k}} = 0$ for a successful scan and ${X_{i,k}} = 1$ for an unsuccessful scan. ${X_o}$ was defined as the target failure rate for successful ultrasound image acquisition and interpretation. In this pilot study, ${X_o}$ was set at 0.02 based on the estimated average success rate of specialist physicians at our institution. A total of 103 consecutive cases were assessed by 16 specialist physicians. Within this dataset, two scanning failures were documented, corresponding to an observed failure rate of approximately 2%. This value was used as a stringent proficiency benchmark rather than as the expected failure rate of novice trainees. The CUSUM value of the AI group or the conventional group at attempt i is defined by

${S_i} = \mathop \sum \limits_{j = 1}^i \mathop \sum \limits_{k = 1}^n {\delta _{i,k}},$

where n represents the total number of trainees in the respective group. A positive slope in the CUSUM curve indicates that the group’s failure rate at that attempt was higher than the expected failure rate, whereas a negative slope indicates a lower failure rate than expected.

The secondary outcomes included the following: (1) Stage-specific success rate: the 15 training cases were divided into three phases (Phase 1: Cases 1–5; Phase 2: Cases 6–10; and Phase 3: Cases 11–15). For each phase, the number of successful scans (*i.e*., successful image acquisition and interpretation, N) and the total number of scans (M) were recorded for each group, and the stage-specific success rate was calculated as N/M × 100%. (2) Time to successful scanning and interpretation, defined as the duration (seconds) from probe placement on the volunteer to image freezing for a successful case. (3) Trainee questionnaire score, calculated as the sum of four Likert-scale questions: (a) Were the content of the training sufficient for your learning? (b) Were you satisfied with your experience with the lessons? (c) Do you feel confident in applying your ability to acquire and interpret images of the sciatic nerve in the popliteal fossa in clinical practice? and (d) do you have the desire to learn more about ultrasound-assisted nerve block? Each question was scored as 1 (“strongly disagree”), 2 (“disagree”), 3 (“neither agree nor disagree”), 4 (“agree”), or 5 (“strongly agree”). (4) Debriefing duration, defined as the time (minutes) from the start of one-to-one debriefing to the end of the conversation. (5) The proportion of trainee speaking time was assessed from the recorded debriefing sessions by two trained coders using a predefined coding protocol; detailed annotation procedures are provided in the Appendix. Inter-rater reliability was assessed using a single-measure consistency intraclass correlation coefficient [ICC(C,1)], yielding an ICC of 0.86 (95% CI [0.78–0.91]) (Appendix Table A1).

### Sample size calculation

No prior study with an identical setting was identified to inform a formal power calculation. Given the nature of this pilot randomized educational study, the trainee sample size was determined pragmatically. We planned to enroll 20 trainees, balancing feasibility (recruitment, supervision, and training resources) with the need to generate preliminary estimates (means/variances and effect sizes) to inform future adequately powered trials. This target aligns with common recommendations for pilot studies and the sample sizes used in several previous studies with partly similar settings (Kollmann-Camaiora et al., 2017; Levy et al., 2024; Park et al., 2019).

The number of training cases was informed by a prior ultrasound education study in which novices typically approached a performance plateau after approximately 8–10 cases (Barrington et al., 2016). Taking into logistical considerations within our institution (teaching time, faculty availability, and scanner access), a total of 15 cases were thus deemed sufficient to construct CUSUM learning curves and capture early performance trends while remaining operationally manageable.

### Statistical analysis

Statistical analyses were conducted using standard statistical methods. For intergroup comparisons, continuous variables with a normal distribution are presented as the mean ± standard deviation (SD) and were compared *via* Student’s *t* test. Continuous variables with a nonnormal distribution are presented as the median (interquartile range [IQR]) and were compared *via* the Mann–Whitney U test or the Kruskal–Wallis test, as appropriate. Categorical variables are expressed as counts (percentages) and were compared *via* the χ^2^ test. A two-sided *P* value < 0.05 was considered statistically significant. The CUSUM curves were plotted for both groups and fitted by a polynomial fitting algorithm. The goodness of fit was assessed *via* the coefficient of determination (*R*^2^), with values closer to 1 indicating a better fit. The model with the highest *R*^2^ value was selected as the optimal fit. The first derivative of the fitted polynomial curve was calculated to identify stationary points at which the slope equaled zero. When more than one stationary point was identified, the local minimum of the fitted CUSUM curve was selected as the learning-curve transition of interest, because it represented the end of the descending CUSUM segment and the transition toward a more stable late-stage trajectory. The preceding local maximum was not considered the relevant learning transition. To account for repeated measurements within trainees, the principal inferential analysis of the scanning outcomes was performed at the participant level. For each trainee, phase-specific success rates and mean successful scanning times were summarized for Cases 1–5, 6–10, and 11–15. Pooled scan-level success proportions (*i.e*., pooled scans from all participants) were reported descriptively only. For debriefing outcomes, the mean debriefing time and mean proportion of trainee speaking time across the 15 sessions were calculated for each trainee.

## Results

### Baseline characteristics

A total of 21 anesthesiology residents and 15 healthy volunteers were recruited and completed the study. Ten residents were allocated to the AI group, and eleven were allocated to the conventional group. The mean ages of the AI and conventional groups were 27 ± 2 (mean ± SD) and 26 ± 2 years, respectively. There were no statistically significant differences between the two groups in terms of age, sex, training grade, or academic qualification. Over the course of the study, the AI group performed a total of 150 scans, and the conventional group performed 165 scans. The baseline characteristics of the residents are summarized in Table 1.

**Table 1:** Baseline characteristics of the trainees.

	AI group	Conventional group	*P* value
Age (years, mean ± SD)	27 ± 2	26 ± 2	0.3776
Sex (Male/total)	5/10	6/11	>0.99
Grade (1 y/2 y)	2/8	2/9	>0.99
Academic qualification (D/ND)	3/7	2/9	0.6351

**Note:**

AI, Artificial intelligence; SD, standard deviation; D, doctoral degree; ND, non-doctoral degree.

### Primary outcome

The CUSUM curve equations were $y = 1.293 + 6.293x - 0.657{x^2} + 0.022{x^3}$ and $y = 2.100 + 4.523x - 0.366{x^2} + 0.009{x^3}$ for the AI and conventional groups, respectively (Fig. 3). The fitted CUSUM curve identified a group-level turning point (TP) at 11.89 attempts in the AI group, corresponding to approximately 12 attempts. In the conventional group, the corresponding TP was at 17.58 attempts, which was outside the observed training range and therefore represented an extrapolative estimate. Thus, no comparable group-level turning point was identified within the 15 observed attempts in the conventional group.

**Figure 3:**
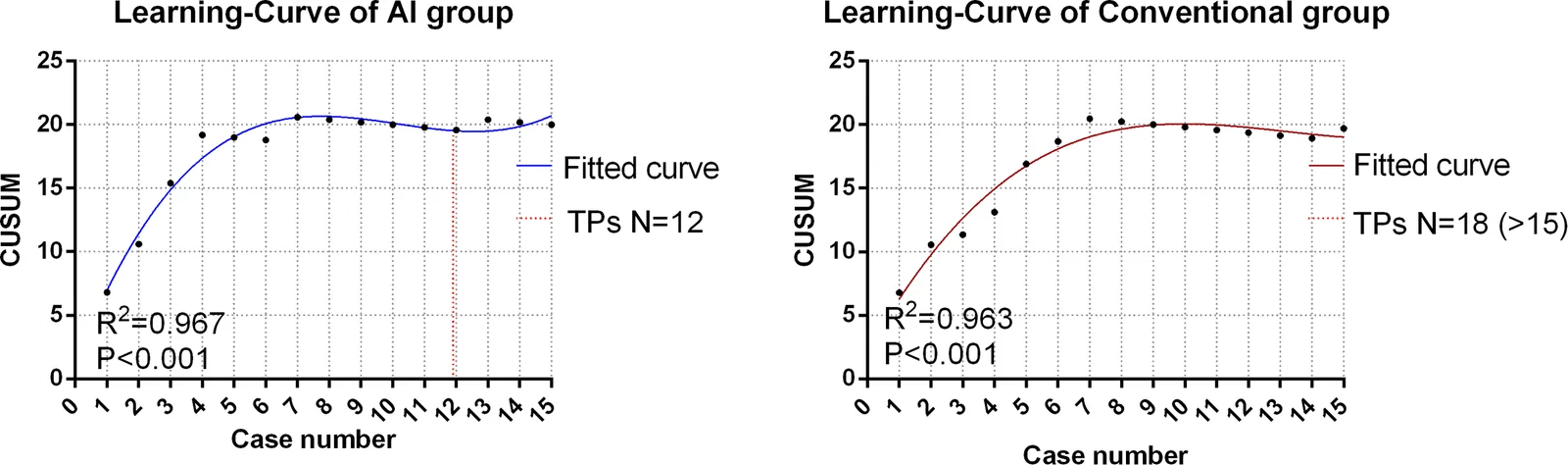
Cumulative sum (CUSUM) learning curves for ultrasound image acquisition and interpretation in the AI-assisted group and the conventional group. The turning points (TPs) were derived from the fitted polynomial curves. The estimated TP in the AI group occurred within the observed 15-attempt training range, whereas the corresponding estimate in the conventional group lay beyond the observed range (approximately 18 attempts) and therefore represented an extrapolative estimate.

### Secondary outcomes

At the participant level, no statistically significant between-group differences in phase-specific success rates were detected in Phase 1 [median (IQR): 60.0% (55, 80) *vs*. 80.0% (40, 80), *P* = 0.6531], Phase 2 [100% (95, 100) *vs*. 100% (80, 100), *P* = 0.6351], or Phase 3 [100% (100, 100) *vs*. 100% (100, 100), *P* > 0.9999]. Successful scanning times also did not differ significantly between groups in Phase 1 [99 (79, 144) *vs*. 97 (85, 158.5) s, *P* = 0.4155] or Phase 2 [62 (57, 79) *vs*. 62 (56, 84) s, *P* = 0.8220], whereas successful scanning time was shorter in the conventional group during Phase 3 [61 (55, 69) *vs*. 54 (46, 58) s, *P* = 0.0207; Table 2]. The pooled scan-level success proportions in the AI and conventional groups were 64% *vs*. 67%, 96% *vs*. 93%, and 98% *vs*. 98% in Phases 1, 2, and 3, respectively (Table S1). With respect to the course experience questionnaire, the AI group reported significantly higher scores than did the conventional group for Question 1 (4.7 ± 0.5 *vs*. 3.6 ± 1.0, *P* = 0.0076), Question 2 (4.9 ± 0.3 *vs*. 3.5 ± 0.8, *P* < 0.0001), and Question 3 (5.0 ± 0.0 *vs*. 4.5 ± 0.7, *P* = 0.0216). There was no significant difference between the groups for Question 4 (*P* > 0.05) (Fig. 4). At the participant level, the mean debriefing time was significantly shorter in the AI group than in the conventional group (7.33 ± 0.34 *vs*. 10.65 ± 0.61 min, *P* < 0.0001), whereas the proportion of trainee speaking time during debriefing was significantly greater in the AI group (69.5% (65.70, 70.55) *vs*. 49.0% (46.70, 49.70), *P* < 0.0001; Table 3).

**Table 2:** Participant-level comparison of phase-specific success rates and successful scanning times between the AI-assisted and conventional groups.

		AI group	Conventional group	*P* value
Phase 1 Case 1–5	Success rate (%)	60.0 (55, 80)	80.0 (40, 80)	0.6531
	Successful scanning time (s)	99 (79, 144)	97 (85, 158.5)	0.4155
Phase 2 Case 6–10	Success rate (%)	100 (95, 100.0)	100 (80, 100.0)	0.6351
	Successful scanning time (s)	62 (57, 79)	62 (56, 84)	0.8220
Phase 3 Case 11–15	Success rate (%)	100 (100, 100.0)	100 (100, 100)	>0.9999
	Successful scanning time (s)	61 (55, 69)	54 (46, 58)	0.0207

**Note:**

Data are presented as median (IQR). AI, Artificial intelligence.

**Figure 4:**
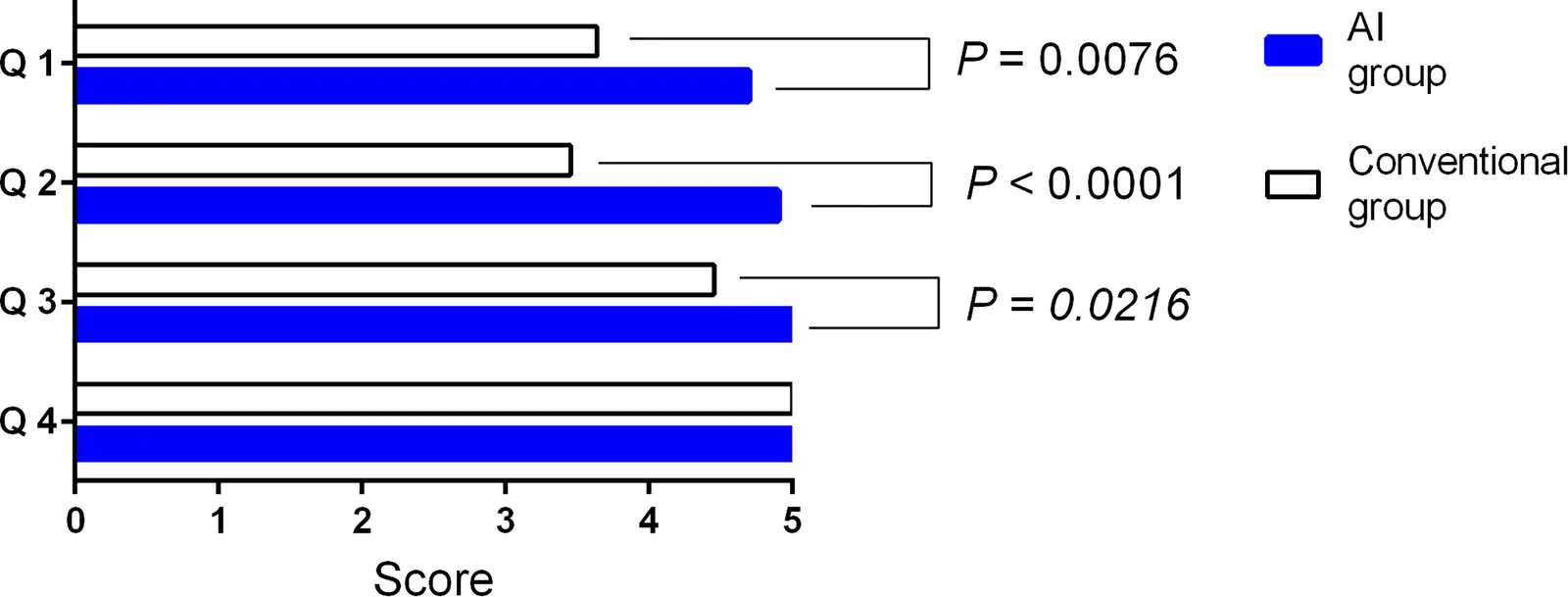
Scores of the post-training course experience questionnaire. The questionnaire assessed perceived adequacy of training content, satisfaction with the learning experience, confidence in applying image acquisition and interpretation skills, and willingness to continue learning ultrasound-assisted nerve block.

**Table 3:** Participant-level summarized debriefing time and proportion of speaking time during debriefing.

	AI group	Conventional group	*P* value
Time of debriefing (min)	7.33 ± 0.34	10.65 ± 0.61	<0.0001
Proportion of speaking time during debriefing (%)	69.5 (65.70, 70.55)	49.0 (46.70, 49.70)	<0.0001

**Note:**

AI, Artificial intelligence. Data are presented as mean ± SD or median (IQR), as appropriate.

## Discussion

In this pilot randomized educational study, we evaluated the role of an AI-assisted ultrasound system in teaching ultrasound image acquisition and interpretation of the sciatic nerve in the popliteal fossa *via* CUSUM learning curves in a group of trainee residents. An exploratory group-level CUSUM TP was identified at approximately 12 attempts in the AI group, whereas no comparable turning point was identified within the 15-attempt training period in the conventional group. We also found that, compared with the conventional group, the AI group had shorter debriefing time, a greater proportion of trainee speaking time, and higher ratings of satisfaction, learning experience, and confidence, suggesting potential benefits for learner experience and debriefing efficiency during early training.

We applied the CUSUM method to quantitatively assess the learning curves of 21 novices. Learning curves are widely used to evaluate skill acquisition, reflecting the rate at which proficiency is achieved over time and serving as an important indicator of training effectiveness (Jung et al., 2021; Moga et al., 2024; Niewodowski & Langton, 2022). The CUSUM approach provides an objective and quantitative description of distinct phases in the learning process (Lin et al., 2023). In our study, the fitted CUSUM curves showed changes in trajectory as training progressed, reflecting improvements in performance during repeated practice. In the rapid ascending phase, most scans were unsuccessful, as trainees encountered challenges in hand‒eye coordination and establishing spatial correspondence between anatomy and ultrasound images (El-Zehiry et al., 2013; Kim et al., 2014), often under the pressure of performance. During this period, they also became familiar with equipment operation and image acquisition procedures. This was followed by the slow ascending phase, during which trainees exhibited marked improvement in technical skills; having mastered basic equipment handling, their focus shifted to refining image acquisition and interpretation. With continued practice, scan accuracy increased and remained high during the later training attempts. Notably, the fitted CUSUM curve for the AI group showed a TP at approximately 12 attempts, which fell within the observed training period and was interpreted as an exploratory group-level transition toward performance stabilization. In contrast, the corresponding TP of the fitted curve for the conventional group was estimated at approximately 18 attempts, beyond the 15 observed training attempts. The within-range TP observed in the AI group may reflect stabilization of image acquisition and interpretation performance during the initial training period. However, both groups achieved high success rates during the later training attempts, with only a limited number of failures reported in either group after the seventh attempt. In addition, the participant-level analysis showed no difference in Phases 1 and 2 but a shorter scanning time in the conventional group during Phase 3. One possible explanation is that, after initial performance stabilization, the additional AI-generated information may impose some cognitive load or interfere with rapid independent judgment; however, this interpretation remains speculative, as these mechanisms were not directly assessed, and the incremental benefit of AI may diminish when baseline performance is already high (Tong et al., 2023). The CUSUM values, calculated through the aggregation of sequential outcomes for all trainees within each group, reinforce the notion that the resulting curve reflects the average learning trajectory of the cohort. These findings should be interpreted in the context of the pilot study design and limited sample size, and the group-level turning points should not be regarded as the number of procedures required for an individual trainee to achieve competence. Nevertheless, the identification of a group-level TP in the AI group may provide a preliminary signal regarding the potential role of AI-assisted ultrasound during early novice training. Future research with larger sample sizes is warranted to validate this preliminary learning curve signal.

While considerable efforts have focused on improving needle visibility through the development of needle-guidance systems and echogenic needles (Brookes et al., 2015; Scholten et al., 2017), these improvements cannot overcome the difficulties in image acquisition and interpretation, which are more common causes of block failure (Swain et al., 2024). Current recommendations for education and training in ultrasound-guided regional anesthesia conceptualize the process into four key steps: (1) image optimization, (2) image interpretation, (3) visualization of needle insertion and injection, and (4) maintenance of needle visibility (Sites et al., 2010). These steps highlight the critical importance in improving image interpretation and recognition compared with the puncture technique itself (Ashken & Thompson, 2020). In practice, novice anesthesiologists may experience difficulties due to gaps in anatomical knowledge (Bowness et al., 2019), inadequate training opportunities (Saltzman et al., 2020), and the susceptibility of human image interpretation to fatigue and errors (Connor, 2019; Drew, Võ & Wolfe, 2013). Furthermore, some patients may exhibit complex anatomy not involved in training sessions, where healthy volunteers are mostly used. To address these challenges, sufficiently trained AI systems offer fatigue-free image recognition and can provide real-time feedback and assistance to novice operators. The WiNerve AI-assisted system, which is based on a deep learning U-Net architecture (Huang et al., 2019), optimizes local anatomical imaging and uses color overlays for structure segmentation. A previous study showed that this AI system can identify target structures of the popliteal sciatic nerve with an accuracy of up to 99% (Bowness et al., 2023a; Cai et al., 2022). For beginners with no prior experience, the WiNerve AI system can highlight key anatomical structures in real time through color overlays, helping them understand the likely location and morphology of these structures in subsequent ultrasound examinations. Moreover, continuous color mapping reduces visual fatigue during image search (Ogura et al., 2017), potentially supporting novice performance during early training and facilitating earlier progression to the needle insertion and therapeutic phases. Overall, these features may have contributed to the within-range CUSUM transition observed in the AI group, reduced supervision time, and greater task-related verbal participation during debriefing. However, because real-time visual feedback is an inherent feature of AI-assisted ultrasound, these findings should be interpreted as reflecting an AI-supported training environment rather than the isolated effect of the AI algorithm alone.

An important feature of our curriculum is the incorporation of introductory sessions on the principles and ethics of AI-assisted ultrasound, where the instructors use ultrasound devices equipped with the AI system during demonstrations. These sessions may have contributed to the favorable teaching outcomes observed in the AI-assisted ultrasound group (Lee et al., 2021). Although our expert panel initially expressed concerns that both “incorrect” and “correct” AI-generated annotations might interfere with novice judgment or divert attention from other anatomical structures (Karmakar et al., 2023), potentially affecting accuracy (Karmakar et al., 2023; Lee et al., 2021), we found no statistically significant differences in accuracy between the two groups at different learning stages. This suggests that the potential interference or cognitive load introduced by the AI system had a minimal impact on learning outcomes in this study, likely owing to the high accuracy of WiNerve in identifying popliteal anatomical structures and the trainees’ strong acceptance and management of AI assistance.

Another interesting finding is that the AI group demonstrated significantly shorter debriefing times and a higher proportion of trainee speaking time than the conventional group. This finding may suggest that AI-assisted visual guidance was associated with more focused post-scan correction and greater task-related verbal participation during debriefing. Such changes may reduce the teaching workload and resource demands and may also reduce the risk of clinical errors by alleviating time pressure and emotional burden on instructors (Heyhoe et al., 2016). In face-to-face teaching, AI may serve as a third-party participant, guiding students through real-time feedback and shifting the learning atmosphere from being “evaluated” to being “supported” (Almegren et al., 2025; Lee, 2024; Liu, Chen & Yao, 2022). Its presence may also help decentralize authority (Cabellos, de Aldama & Pozo, 2024; Velli & Zafiropoulos, 2024; Zhou et al., 2025), thereby reducing student anxiety and encouraging open discussion and collaborative problem solving. These dynamics likely contributed to the AI group’s higher level of satisfaction, better learning experience, and greater confidence. From an educational perspective, AI-assisted ultrasound may be particularly useful as an adjunctive tool when direct instructor availability is limited because it can provide real-time visual guidance during image acquisition and support novice trainees during the early stage of anatomical recognition. However, these questionnaire-based outcomes should be interpreted as indicators of trainee perceptions and learning experience rather than evidence of superior clinical performance. Further studies are warranted to validate these contributing factors involved in AI-assisted teaching.

This study has several limitations. First, since this was a single-center pilot randomized educational study with a small sample size, and no formal power calculation was performed, the statistical robustness of the findings is limited, and the results should be interpreted as preliminary and hypothesis-generating rather than confirmatory. In addition, the CUSUM analysis was based on a single institutional target failure rate of 2%, and sensitivity analyses using alternative thresholds were not performed. Blinding of the outcome assessors was also not feasible because the AI-generated anatomical overlays were visible in the recordings, and formal inter-rater reliability for the image-success assessment was not assessed; therefore, the possibility of assessment bias and measurement variability cannot be fully excluded. In addition, several outcomes were subjective and based solely on the acquisition and interpretation of WiNerve-assisted popliteal sciatic nerve ultrasound images in a single, restricted scenario using only healthy volunteers. Therefore, the findings may not fully reflect actual clinical practice or the teaching of more complex procedures involving patients with variable anatomy. Second, there was an imbalance in the training and feedback structure between the two groups. The AI group received additional instruction on the principles and ethical considerations of AI-assisted ultrasound and real-time visual feedback through AI-generated anatomical overlays, whereas the conventional group received corrective feedback only after image acquisition during debriefing. Therefore, the observed effects should be interpreted as reflecting AI-assisted ultrasound as a complete educational intervention, rather than the isolated effect of the AI algorithm alone. Third, all 21 participating residents were from the same hospital in a single region. Differences in ultrasound experience, practice patterns, and cultural factors across regions may lead to different results, indicating the importance of conducting larger multicenter studies among trainees with different skill levels. Fourth, the study was conducted in a nonclinical setting, and its findings may differ in real-world clinical contexts. This limitation underscores the need for further investigation into the real-world clinical performance of anesthesiologists in ultrasound-guided regional nerve blocks with and without AI-assisted ultrasound systems. Furthermore, the AI intervention included both real-time AI-generated overlays and mandatory instruction on the principles and ethics of AI-assisted ultrasound; therefore, the independent contribution of the AI algorithm cannot be determined. The questionnaire was not validated, and trainee-reported responses may have been influenced by novelty effects, expectation bias, and ceiling effects; these findings should therefore be regarded as exploratory perceptions rather than validated measures of educational effectiveness. Finally, trainee performance was assessed while AI assistance remained active, without unaided post-training or retention assessment; therefore, whether the observed performance differences translate into durable or transferable skill acquisition remains unknown. Overall, the interpretation of the results should be approached with caution, and further studies are necessary to validate these findings.

## Conclusions

In this pilot randomized educational study, AI-assisted ultrasound training for trainee anesthesiologists was associated with a group-level CUSUM TP identified within the observed training range, shorter debriefing time, a greater proportion of trainee speaking time during debriefing, and higher trainee-reported satisfaction, learning experience, and confidence. However, no statistically significant differences were detected in stage-specific success rates. These findings suggest that AI-assisted ultrasound may be a useful adjunct for learning nerve block image acquisition and interpretation. Adequately powered studies are needed to determine whether these preliminary findings translate into improved procedural performance in clinical practice.

## Supplemental Information

10.7717/peerj.21877/supp-1 Supplemental Information 1 Raw data.

10.7717/peerj.21877/supp-2 Supplemental Information 2 Speaking time.

10.7717/peerj.21877/supp-3 Supplemental Information 3 Pooled phase-specific scan success rates of the AI-assisted and conventional groups.

## Additional Information and Declarations

### Data Availability

The following information was supplied regarding data availability:

The data is available in the Supplemental Files.

The data is available at Zenodo: Wang et al. (2026). Artificial Intelligence-Assisted Ultrasound for Teaching Popliteal Fossa Sciatic Nerve Block to Novice Anesthesiologists-preliminary (Version version-01) [Figure]. Zenodo. https://doi.org/10.5281/zenodo.20150417.
