# Supplementary material for: Learning curve analysis of artificial intelligence-assisted ultrasound for teaching popliteal fossa sciatic nerve block to novice anesthesiologists: a pilot randomized educational study
Source: PeerJ. 2026 Nov 12;14:e21877. doi: 10.7717/peerj.21877 (PMC13770415; doi:10.7717/peerj.21877)
Supplement: Supplementary file 1 [file peerj-14-21877-s002.docx]

**Appendix**

Methods for Measuring Trainee Speaking Time

· **Recording.** All instructional sessions were recorded utilizing the automated audio/video recording system of the simulation training center. This process was overseen by a teaching-center assistant who was not involved in the subsequent analysis.

· **Coders and Protocol.** Two trained coders independently annotated the recordings in accordance with a predefined coding protocol. The average of their measurements was utilized for analysis. Prior to formal coding, both coders underwent a calibration phase: they reviewed the comprehensive coding scheme, practiced on 2–3 sample recordings, discussed any discrepancies, and achieved consensus on calibration.

· **Blinding.** All audio files were anonymized and devoid of group-identifying information; the coders were blinded to group allocation (AI group versus conventional group).

· **Annotation Rules.** The recordings were annotated segment by segment using the EUDICO Linguistic Annotator (ELAN), with the start and end time of each segment marked and the total duration summed.

Valid trainee speech was defined as task-related verbal output, which included describing ultrasound image structures, verbalizing operational reasoning, posing task-related questions, articulating one's own reasoning and judgments, and responding to the instructor's task-related inquiries (including brief task-related yes/no responses, even if lacking fluency).

Excluded from timing were all instructor speech (including instructions, feedback, questions, corrections, and explanations), extended silences and pauses, meaningless fillers (e.g., "um," "uh"), small talk unrelated to the scanning task, and mechanical repetition of the instructor's statements without new content. Boundary rules stipulated that for overlapping speech, only segments distinctly attributable to the trainee were timed; within a single continuous speaking turn, pauses of ≤2 seconds were merged, while pauses exceeding 2 seconds resulted in truncation of timing. Simple "yes/no" task-related responses were counted, whereas purely tonal acknowledgments (e.g., "mm-hmm," "oh") were excluded.

· **Discrepancy Resolution.** In instances where the results of the two coders significantly differed (>2 minutes), the coding protocol was re-examined, and the disputed cases were jointly reviewed with a third adjudicator.

**Appendix Table A1. Inter-rater reliability assessment of trainee speaking-time coding**

| **Sample No.** | **Coder A speaking time (s)** | **Coder B speaking time (s)** |
| --- | --- | --- |
| 1 | 166 | 104 |
| 2 | 99 | 165 |
| 3 | 102 | 186 |
| 4 | 161 | 145 |
| 5 | 190 | 122 |
| 6 | 168 | 192 |
| 7 | 255 | 105 |
| 8 | 209 | 151 |
| 9 | 141 | 147 |
| 10 | 148 | 182 |
| 11 | 216 | 264 |
| 12 | 201 | 129 |
| 13 | 80 | 100 |
| 14 | 352 | 488 |
| 15 | 164 | 196 |
| 16 | 172 | 98 |
| 17 | 311 | 409 |
| 18 | 130 | 230 |
| 19 | 140 | 130 |
| 20 | 125 | 115 |
| 21 | 201 | 159 |
| 22 | 234 | 126 |
| 23 | 335 | 265 |
| 24 | 305 | 271 |
| 25 | 371 | 301 |
| 26 | 332 | 388 |
| 27 | 315 | 357 |
| 28 | 246 | 330 |
| 29 | 304 | 368 |
| 30 | 560 | 520 |
| 31 | 297 | 423 |
| 32 | 146 | 166 |
| 33 | 214 | 236 |
| 34 | 179 | 181 |
| 35 | 84 | 108 |
| 36 | 283 | 341 |
| 37 | 409 | 431 |
| 38 | 421 | 299 |
| 39 | 204 | 66 |
| 40 | 252 | 252 |
| 41 | 319 | 281 |
| 42 | 120 | 240 |
| 43 | 80 | 136 |
| 44 | 491 | 517 |
| 45 | 642 | 528 |
| 46 | 537 | 615 |
| 47 | 237 | 243 |
| 48 | 375 | 225 |
| 49 | 465 | 459 |
| 50 | 270 | 234 |
| 51 | 391 | 509 |
| 52 | 231 | 129 |
| 53 | 316 | 284 |
| 54 | 271 | 257 |
| 55 | 408 | 456 |
| 56 | 234 | 294 |
| 57 | 347 | 373 |
| 58 | 340 | 284 |
| 59 | 256 | 140 |
| 60 | 470 | 466 |
| 61 | 572 | 520 |
| 62 | 349 | 323 |
| 63 | 282 | 243 |

Of 315 recorded debriefing sessions, 20% (n = 63) were randomly sampled and independently coded by two coders. Inter-rater reliability: ICC(C,1) = 0.859 (95% CI, 0.777-0.912). ICC(C,1), single-measure consistency intraclass correlation coefficient; CI, confidence interval.
