# Supplementary material for: Learning curve analysis of artificial intelligence-assisted ultrasound for teaching popliteal fossa sciatic nerve block to novice anesthesiologists: a pilot randomized educational study
Source: PeerJ. 2026 Nov 12;14:e21877. doi: 10.7717/peerj.21877 (PMC13770415; doi:10.7717/peerj.21877)
Supplement: Supplementary file 2 [file peerj-14-21877-s003.docx]

**Supplementary Table S1. Pooled phase-specific scan success rates of the AI-assisted and conventional groups.**

|  | AI group | Conventional group |
| --- | --- | --- |
| Total successful scans in Phase 1 (N(%)) | 32(64) | 37(67) |
| Total successful scans in Phase 2 (N(%)) | 48(96) | 51(93) |
| Total successful scans in Phase 3 (N(%)) | 49(98) | 54(98) |

AI, artificial intelligence.
